# Do BRAF inhibitors select for populations with different disease progression kinetics?

**DOI:** 10.1186/1479-5876-11-61

**Published:** 2013-03-08

**Authors:** Paolo Antonio Ascierto, Ester Simeone, Antonio Maria Grimaldi, Marcello Curvietto, Assunta Esposito, Giuseppe Palmieri, Nicola Mozzillo

**Affiliations:** 1 Unit of Melanoma, Cancer Immunotherapy and Innovative Therapy, Department of Melanoma, Istituto Nazionale Tumori Fondazione “G. Pascale”, Naples, Italy; 2 Unit of Cancer Genetics, Institute of Biomolecular Chemistry, C.N.R, Sassari, Italy; 3 Unit of Melanoma and Sarcoma Surgery, Department of Melanoma, Istituto Nazionale Tumori Fondazione “G. Pascale”, Naples, Italy

## Abstract

Ipilimumab, an anti-CTLA-4 monoclonal antibody, has been shown to improve overall survival in patients with metastatic melanoma. Preliminary data suggest that patients who fail BRAF inhibitor treatment experience a very rapid progression of disease. Such selectivity for more rapid disease progression may mean these patients do not receive the same benefit from subsequent treatment with ipilimumab as patients without prior BRAF inhibitor treatment. The current challenge is focused on how to identify and approach the two populations of fast and slow progressors and recent hypothesis suggest that treatment choice could be guided by baseline risk factors. However, no data have yet defined which the best sequence is and more research is needed to identify predictors of response in patients with metastatic melanoma to help guide whether a BRAF inhibitor or ipilimumab should be used first in sequential therapy.

## Commentary

The recent availability of new drugs for the treatment of patients with metastatic melanoma has profoundly changed the therapeutic approach to a disease with previously poor prognosis, in which no drug had increased survival in randomized trials for over 30 years.

However, the introduction of novel drugs into clinical practice can rapidly generate new data, offering extra insights into their therapeutic use. This is currently happening in metastatic melanoma, where recent experience has indicated that around half of patients receiving BRAF inhibitors do not gain the same benefit from subsequent treatment with ipilimumab as BRAF inhibitor treatment-naïve patients. This may be a result of BRAF inhibitor drug resistance activating some process of cellular/metabolic escape, thus selecting a more aggressive disease.

Ipilimumab has been shown to improve overall survival in approximately 80% of patients with metastatic melanoma who have not received prior therapy with BRAF inhibitors [1]. The remaining 20% who did not respond appeared to be those who received only one or two doses of ipilimumab. Consistent with this, analysis of around 900 patients who were treated in Italy within a compassionate expanded access program revealed that approximately 23% of patients were not able to continue beyond the second ipilimumab administration [2]. These findings are in agreement with its mechanism of action, since by acting as an “activator” of the immune system and not as a cytotoxic drug, ipilimumab requires a latency period in order to show efficacy. Both these datasets included patients regardless of BRAF mutational status, with mutation analysis not being performed in all patients due to the absence of drugs against this target at the time. However, as the population with this mutation corresponds to approximately half of the total, it is likely to assume that wild-type and mutated patients were equally represented.

Although preliminary, recent data suggest that patients who fail BRAF inhibitor treatment experience a very rapid evolution and progression of disease. The BRIM2 study reported that in 16 of 39 patients (41%) who died as a result of disease progression, death occurred within 28 days after the last administration of the drug [3]. Similarly, in the BRIM3 study, 22 of 42 patients (52%) treated with vemurafenib died during the course of the study within 28 days after the last administration, mainly due to disease progression [4].

In a retrospective analysis by our group, 12 of 28 patients (43%) treated with a BRAF inhibitor had rapid disease progression meaning subsequent treatment with ipilimumab was limited to only one or two administrations and could not be completed [5]. An ECOG PS of 1, LDH level ≥1.10 times the upper limit of normal (ULN) and the presence of brain metastases were all associated with not completing the ipilimumab induction regimen. Similarly, Ackerman et al. reported that approximately 50% of patients who received ipilimumab after progression on vemurafenib died within 4 months [6], while the Royal Marsden Hospital reported that 38% of patients who failed on vemurafenib were not able to complete a second line treatment due to the rapid progression of disease [7]. In addition, in the compassionate use program of ipilimumab in Italy, it was observed that 41% of 54 patients who had received prior treatment with BRAF inhibitors did not receive a third dose of ipilimumab [2].

In conclusion, although these data are still preliminary and obtained from limited numbers of patients, taken together they suggest that around half of patients (range 38–52%) that fail treatment with a BRAF inhibitor have a much more rapid disease progression than those who have not received BRAF inhibitor therapy (Table 1). The potential for Ipilimumab to provide a clinical benefit in these patients is limited since they are unable to receive more than 1 or 2 cycles of the drug.

**Table 1:** Different evidences of rapid progression disease after BRAF inhibitors treatment

**Experience**	**Patients sample (n)**	**Percentage of patients with a rapid disease progression kinetics**
BRIM-2 [3]	39	41%
BRIM-3 [4]	42	52%
Ascierto et al. [5]	28	43%
Ackerman et al. [6]	32	50%
Italian ipilimumab EAP [2]	54	41%
Fisher et al. [7]	42	38%

EAP: Expanded access program.

As a consequence, the BRAF mutated patients should be approached, from a therapeutic point of view, considering the 2 groups: the slow and the fast progressors.

The first group includes patients that, due to the kinetics of their illness, can be treated with both BRAF inhibitors and ipilimumab. In a recent publication, Jang and Atkins [8] suggest treatment with ipilimumab first in patients with indolent disease, while our hypothesis is that patients with maximum one risk factor can benefit from receiving BRAF inhibitor as the first part of their sequential treatment regimen [5].

However, no data have yet shown which the best sequence is: considering the drug schedule, it could be convenient to start with ipilimumab.

On the other hand, it is much more important to define the correct approach for fast progressors. Considering that pretreatment with BRAF inhibitors increases (double) the numbers of this fast progressors population, these patients are at high risk of not receiving a subsequent treatment with ipilimumab. This is the main reason why, in our previous report, we suggested starting with ipilimumab; moreover, our preliminary data showed that even patients with negative prognostic factors were able to receive ipilimumab and subsequent BRAF inhibitor upon progression. In our proposed algorithm, rapid or slow progressions of disease were evaluated according to baseline factors and, although further data are needed, in the absence of prospective clinical data to guide the choice of treatment sequence, this could help in the treatment decision (Table 2).

**Table 2:** Proposed baseline factors to identify slow and fast progressors

**Slow progressor**	**Fast progressor**
PS 0	PS 1
Normal LDH	Elevated LDH
Absence of brain metastasis	Presence of brain metastasis

PS = Performance Status.

The next challenge will be to find some predictive markers that could help to identify the two populations, thus guiding whether a BRAF inhibitor or ipilimumab should be used first in sequential therapy. Further information could come from molecular studies performed on biopsy at baseline and progression in patients treated with B-Raf inhibitors. In the future, combination or sequential approaches, with BRAF plus MEK or PI3K inhibitors and different immunomodulating antibodies (PD-1, anti-PD-L1) could increase the benefit for these patients [9].

## Competing interests

PAA has served as a consultant and advisor for Bristol Myers Squibb, Merck Sharp & Dohme, and Roche-Genentech, GlaxoSmithKline, Amgen, Celgene, Medimmune and Novartis; he received honoraria from Bristol-Myers Squibb, Merck Sharp & Dohme and Roche-Genentech. ES received honoraria from Bristol-Myers Squibb. All remaining Authors have no competing interests.

## Authors’ contributions

PAA, ES, AMG, MC, AE, GP, and NM: 1) made intellectual contributions and participated in the acquisition, analysis and interpretation of data; 2) have been involved in drafting the manuscript; and 3) have given final approval of the version to be published.
